# Cholestase anictérique: une manifestation hépatique rare d’hyperthyroïdie

**DOI:** 10.11604/pamj.2019.34.215.19988

**Published:** 2019-12-30

**Authors:** Dayssem Khelifi, Ibtissem Ben Nacef, Imen Rojbi, Nadia Mchirgui, Karima Khiari

**Affiliations:** 1 Université de Tunis El Manar, Faculté de Médecine de Tunis, Service d’Endocrinologie-Diabétologie, Hôpital Charles Nicolle, Tunis, Tunisie

**Keywords:** Cholestase anictérique, maladie de Basedow, hyperthyroïdie, Anicteric cholestasis, Graves’ disease, hyperthyroidism

## Abstract

Les manifestations cliniques et les atteintes viscérales décrites au cours des affections de la thyroïde sont nombreuses et variées. Toutefois, de rares cas de manifestations hépatiques sont rapportés dans la littérature. Nous rapportons le cas d'un patient âgé de 50 ans chez qui le diagnostic d'une maladie de Basedow sans goitre ni manifestation oculaire a été retenu. Le bilan biologique de surveillance a permis la découverte d'une cholestase anictérique. Devant un bilan étiologique négatif et une normalisation du bilan hépatique après un traitement bien conduit de l'hyperthyroïdie, le diagnostic d'une atteinte hépatique secondaire à une maladie de Basedow a été retenu. Le but de ce travail est de rapporter un nouveau cas d'une manifestation hépatique rare (cholestase anictérique) au cours de l'hyperthyroïdie: maladie de Basedow.

## Abstract (en)

Clinical manifestations and visceral involvements described in patients with thyroid disease are many and varied. However, cases of patients with hepatic manifestations have been rarely reported in the literature. We here report the case of a 50 year old patient diagnosed with Graves' disease without a goiter or ocular manifestations. Follow-up laboratory testing led to the detection of anicteric cholestasis. Investigations of the potential causes were negative and given the normalization of the liver parameters after appropriate treatment of hyperthyroidism, the diagnosis of hepatic involvement secondary to Graves' disease was retained. The purpose of this study was to report a new case of rare hepatic manifestation (cholestasis anictérique) in a patient with hyperthyroidism: Graves' disease.

## Introduction

Le foie joue un rôle important dans le métabolisme des hormones thyroïdiennes, et celles-ci ont également un rôle important dans la fonction hépatique normale [1]. Par conséquent, il n'est pas surprenant que la dysfonction hépatique soit observée chez les patients atteints de maladie thyroïdienne [2]. Ceci a été décrit pour la première fois par Habershon en 1874 [3]. Des anomalies non spécifiques du bilan hépatique pourraient accompagner l'évolution clinique de l'hyperthyroïdie, entraînant une élévation des enzymes hépatiques et de la bilirubine [4]. Des formes cliniques atypiques de la maladie de Basedow, principale cause des hyperthyroïdies, ont été décrites, notamment de rares manifestations hépatiques [5]. Le but de ce travail est de rapporter un nouveau cas d'une manifestation hépatique rare (cholestase anictérique) au cours de l'hyperthyroïdie: maladie de Basedow.

## Patient et observation

Un patient âgé de 50 ans, consulte pour amaigrissement contrastant avec un appétit conservé, palpitations, hypersudation et thermophobie. L'examen clinique trouve un patient tachycarde à 132 bpm, une hypertension artérielle (HTA) systolo-diastolique, indice de masse corporelle (IMC) à 1.15 kg/m^2^, thyroïde non palpable et pas d'exophtalmie. Le diagnostic d'hyperthyroïdie liée à une maladie de Basedow a été retenu devant thyroid stimulating hormone (TSH)<0.05µUI/ml (0.35-4.98), free thyroxine (FT4)=1.98ng/dl (0.7-1.48) et des anticorps anti-récepteurs de la TSH étaient positifs à 20.34UI/L (1.75UI/L). Un traitement par benzylthiouracyl (BTU) 300mg/jour et propanolol 40mg/j a été instauré. Deux mois plus tard, un bilan biologique de contrôle a révélé la persistance de l'hyperthyroïdie et a permis la découverte d'une cholestase anictérique avec des gamma-glutamyl-transpeptidase (GGT) à dix fois la normale, des phosphatases alcalines (PAL) à deux fois la normale associés à des taux de bilirubine, et transaminases normaux. Une enquête étiologique a été ainsi entamée: l'échographie abdominale était normale, les sérologies virales B, C étaient négatives. La recherche des anticorps (AC) anti-mitochondries, anti-muscles lisses, anti-liver kidney microsome type 1 (anti-LKM1) et anti-nucléaires était négative. La bili-imagerie par résonance magnétique (bili-IRM) montrait l'absence de dilatation des voies biliaires intra et extra hépatiques. Malgré l'arrêt du BTU, une majoration de la cholestase a été notée. Le patient a ainsi bénéficié d'une cure d'irathérapie à l'iode 131 (13mCi). L'évolution fut marquée par une nette amélioration clinique, la disparition de la cholestase et la survenue 4 mois après d'une hypothyroïdie post irathérapie, substituée par lymphocytes T auxiliaires (LT4) à la dose de 100ug/j, permettant l'obtention d'une euthyroidie (Tableau 1).

**Tableau 1:** Bilan biologique de surveillance

	(2 mois après début du traitement : BTU)	Initialement Après l’arrêt du BTU	Une semaine après I’irathérapie (I*131)	Quatre mois après I’irathérapie	Après le traitement substitutif	Valeurs usuelles
**FT4 (ng/dL)**	1.69	---	1.65	<0.4	---	0.7-1.48
**TSH (**µ**UI/mL)**	<0.05	---	<0.05	30.33	2.58	0.35-4.98
**GGT (U/L)**	679	745	597	44	31	10-64
**PAL (U/L)**	323	383	265	95	83	40-150
**ASAT(U/L)**	33	33	---	9	11	5-34
**ALAT(U/L)**	61	67	---	8	9	6-55
**Bilirubine totale (**µ**mol/L)**	10.4	7.5	5.7	7	6.6	3.4-20

## Discussion

Les interactions entre le foie et la thyroïde sont nombreuses. Le foie joue un rôle important dans le métabolisme des hormones thyroïdiennes [1], et celles-ci ont également un rôle important dans le maintien d'une fonction hépatique normale et dans le métabolisme de diverses substances, comme la bilirubine et les acides biliaires [1]. Selon la littérature, l´atteinte hépatique au cours de l'hyperthyroïdie est présente dans 37 à 78% des cas [6]. Elle est le plus souvent asymptomatique. Notre patient a présenté une maladie de Basedow sans dysfonction cardiaque. Le bilan biologique de surveillance a permis la découverte d'une cholestase anictérique, sans élévation des taux de la bilirubine ni des autres enzymes hépatiques. L'examen clinique n'a pas mis en évidence un ictère ni d'hépatomégalie. Le bilan étiologique était négatif. L'atteinte hépatique au cours de l'hyperthyroïdie, peut se manifester par un prurit. À l'examen clinique, un ictère, une hépatomégalie et/ou une splénomégalie peuvent être observés [7]. En l´absence de décompensation cardiaque l´ictère est objectivé dans environ 5 à 11% des cas [6]. La fréquence des symptômes cliniques varie selon les études. Sur le plan biologique, l'anomalie la plus fréquente est une élévation de l'activité sérique des phosphatases alcalines, observée dans 64% à 70% des cas [6,8]. Elle est généralement secondaire à une élévation de la fraction osseuse de l'enzyme [6,9]. Les GGT sont élevées dans 17% des cas [5] et les Alanine transaminase (ALAT) dans 28 à 37% des cas [6, 8, 9].

Il n'est pas clair si ces modifications hépatiques résultent d'un effet direct de la thyrotoxicose ou sont causées par des affections associées telles qu'une insuffisance cardiaque congestive, une hépatite auto-immune, virale ou toxique, une infection ou même un état dedénutrition, pouvant elles-mêmes conduire à un dysfonctionnement hépatique [1,2]. Les traitements antithyroïdiens, en particulier le propylthiouracile (PTU), peuvent entraîner une perturbation du bilan hépatique: une élévation de l'activité de l'ALAT est observée chez environ 30% des malades traités. Des cas d'hépatite sévère ont également été rapportés [10]. Les effets secondaires apparaissent généralement au cours des trois premiers mois du traitement, mais ils peuvent également apparaître beaucoup plus tard au cours du traitement [1]. Le traitement par antithyroïdiens de synthèse entraine une évolution favorable malgré les effets délétères hépatiques rares mais avérés des thionamides [5]. Une irathérapie ou une thyroïdectomie peuvent aussi être proposées dans certaines situations [5]. Il est à noter que ces anomalies sont réversibles et qu'elles disparaîtront au fur et à mesure que l'état euthyroïdien sera restauré après le traitement [1]. Dans notre cas, malgré l'arrêt du BTU, une majoration de la cholestase a été notée. L'hypothèse d'une atteinte hépatique liée aux effets indésirables des antithyroïdiens a été exclue. Le patient a ainsi bénéficié d'une cure d'irathérapie à l'iode 131 (13mCi). L'évolution fut marquée par une nette amélioration clinique, la disparition de la cholestase et la survenue 4 mois après d'une hypothyroïdie post irathérapie qui a été substituée. Devant un bilan étiologique négatif et une normalisation du bilan hépatique après un traitement bien conduit de l'hyperthyroïdie, le diagnostic d'une atteinte hépatique secondaire à une maladie de Basedow a été retenu.

## Conclusion

La cholestase anictérique est une manifestation hépatique rare au cours de la maladie de Basedow, souvent asymptomatique. Toutefois, une perturbation de l'enzymologie hépatique est couramment observée, ainsi, devant toute cholestase non étiquetée une hyperthyroïdie doit être recherchée.

## Conflits d’intérêts

Les auteurs ne déclarent aucun conflit d'intérêts.
